# Constitution of the American Dental Association

**Published:** 1869-05

**Authors:** 


### CONSTITUTION.

---

### ARTICLE I.

Name.—This organization shall be known by the name of the American Dental Association.

### ARTICLE II.

Objects.—The objects of this Association shall be, to cultivate the science and art of Dentistry, and all its collateral branches; to elevate and sustain the professional character of Dentists, to promote among them mutual improvement, social intercourse and good feeling, and collectively to represent and have cognizance of the common interests of the Dental Profession.

### ARTICLE III.

SECTION 1.—*Membership. Number of delegates from local Societies.*—Each local society may send one for every five of its active members, and each Dental College one from its faculty as delegates to this Association, upon complying with the requirements of its Constitution, but no society shall be entitled to representation that does not adopt or substantially recognize its code of ethics.

SEC. 2.—*Expelled members.*—Any member of a State or local society, being expelled from the same, shall cease to be a member of this Association from the date of such expulsion.

SEC. 3.—*Members to be of two Classes.*—The members of this Association shall be of two classes, delegates and permanent members, having equal rights and privileges.

SEC. 4.—*Delegates.*—All delegates shall be practitioners of Dentistry; they shall receive their appointment only from permanently organized Dental Societies and Dental Colleges, having definite conditions of membership other than pecuniary, which shall have been approved by the Executive Committee, and a copy of which shall be in the hands of the Recording Secretary of this body.

SEC. 5.—*Permanent Members.*—Permanent members shall consist of those who, having served one year as delegates, and complied with the requirements of the Association, shall signify to the Treasurer a desire for permanent membership.

SEC. 6.—*Signing Constitution.*—Each new member before voting or speaking on any subject before the meeting, shall sign these regulations, inscribing his name, and the title and location of the institution from which he receives his appointment.

SEC. 7.—*Dues.*—Five dollars shall be paid to the Treasurer by each member yearly, as dues, before he can speak or vote on any subject before the Association, and in case the dues remain unpaid at the close of the second annual meeting thereafter, the name of any such delinquent shall be erased from the list of members without further action.

SEC. 8.—*Giving name on rising.*—No one shall be permitted to address the Association before giving his name and residence, which shall be distinctly announced from the chair, nor shall he speak more than twice, nor longer than 15 minutes in all, unless by consent of the Association.

### ARTICLE IV.

*Time of Meetings.*—The regular meetings of the Association shall be held annually, and commence on the first Tuesday in August. The place of meeting shall be determined each year by vote of the Association.

### ARTICLE V.

SECTION 1.—*Officers. How elected.*—The officers of this Association shall be a President, two Vice-Presidents, Corresponding Secretary, Recording Secretary, Treasurer, and the Executive Committee, who shall be elected by ballot without nomination. The two names, or in case of a tie, the three names having the greatest number of votes, shall be the sole nominees in case of failure to elect on the first ballot, and a majority of all the votes cast for those names, shall be necessary to a choice.

Each officer, except as otherwise provided, shall hold his appointment for one year, or until another is elected to succeed him.

SEC. 2.—*President, duties of.*—The President shall preside according to Parliamentary usage, as laid down in Cushing's Manual, and the rules of order adopted by this Association

Sec. 3.—*Vice-President.*—In the absence of the President, one of the Vice-Presidents shall perform the duties of the office, and in the absence of these officers, a chairman *pro tem.*, shall be appointed by acclamation.

Sec. 4.—*Corresponding Secretary.*—The Corresponding Secretary shall attend to the correspondence of the Association with the Societies therein represented, and to correspondence with other scientific bodies as may be desirable.

Sec. 5.—*Recording Secretary.*—The Recording Secretary shall keep accurate minutes of the proceedings of the Association, preserve the archives and unpublished documents, and attend to the other duties that pertain to his office. He shall be *ex-officio* chairman of the Publication Committee, and shall see that due notice is given of the time and place of meeting of the Association.

Sec. 6.—*Executive Committee.*—The Executive Committee shall be the business committee of the Association, to whom shall be referred all business not otherwise specially provided for. They shall report at each meeting, under the proper head their doings for the current year.

*Sub-Committees. 1st Division, Committee of Arrangements.*—This Committee shall be composed of nine members, three of whom shall act as the Committee of Arrangements, and shall procure suitable accommodations for the meetings and clinics, and for the exhibition and examination of appliances, of which they shall have the charge. These three shall, if practicable, reside at or near the place at which the Association is to hold its next annual meeting; and shall attend generally to the wants of the Association during its session.

*2d Division. Credentials and Auditing Committee.*—Other three shall examine and verify the credentials and qualifications of members, and shall also be the auditing committee for that year.

*3d Division. Committee on Nominations and Volunteer Essays.*—The remaining three shall examine all volunteer essays, and before they are read to the Association shall give their approval, in order that time may not be taken up by unworthy or irrelevant matter.

They shall report names for the Standing Committees for the ensuing year, and the names of places for the next meeting.

*Publication Committee, how appointed.*—Immediately after the election of new members to the Executive Committee, two from that committee shall be appointed by the President to act in conjunction with the Recording Secretary, as the Publication Committee. They shall be authorized to employ a competent reporter to furnish an accurate report of the proceedings of each meeting.

*Instructions to the Publication Committee.*—They shall superintend the publication and distribution of such portions of the transactions as the Association may direct, or the committee judge to be of sufficient value. They shall specify in the annual report of this committee the character and cost of the publications of the Association during the year, and the number of copies still on hand.

*Disclaimer.*—This committee is hereby instructed to print at the beginning of each volume of the transactions, the following disclaimer, viz.: "The American Dental Association, although formally accepting and publishing the reports of the various Standing Committees, and the essays read before the Association, holds itself wholly irresponsible for the opinions, theories, or criticisms therein contained, except when otherwise decided by special resolution."

*Reports of sub-committees* —Each of these sub-committees shall report from time to time as may be necessary, to the Executive Committee as a whole, who shall decide upon such report if possible, but in case of their inability to decide upon any matter, it shall be brought before the Association for its decision as early as practicable.

*Classification of Executive Committee.*—At the first election of this committee, the first three shall be elected to serve three years, the second three for two years, and the last three for one year, and thenceforward three new members shall be elected each year by ballot, to serve three years, and in case of the absence of any member of this committee, his place may be temporarily filled by the remaining members of the committee.

*Meetings of Executive Committee.*—The Executive Committee shall, if possible, meet for consultation and arrangement of their respective duties, on the day preceding the annual meeting of the Association, and shall meet for the examination of credentials, the arrangement of appliances, and the reception of papers, and such other business as may properly come before them, at 8 A. M., on the day of the annual meeting.

*Sub-division of Executive Committee.*—They shall also meet after the election of new members to the committee, to choose their own chairman and Secretary, and to divide themselves into three sub-committees as hereinbefore provided, and for the purposes described.

*Chairman may convene the Committee.*—The chairman of this committee may at his own discretion, summon the members thereof to a meeting, at any suitable hour during the sessions of the Association.

SEC. 7.—*Treasurer, to attend to receive dues at* 8 *A. M.*—The Treasurer shall hold all the monies belonging to the Association, and shall keep an accurate account as between the society and its members. He shall attend the meeting of the Executive Committee at 8 A. M., on the day of the Annual Meeting, to receive dues, and shall attend at roll call to verify the list of names, checking all such as are in arrears for dues, he shall restore such names upon payment of dues within the legal limits, and shall notify all whose names may be erased from the list of membership. He shall pay the drafts of the President countersigned by the Secretary upon vote of the society only, and shall report to the Executive Committee where his accounts shall be audited, and by whom his report shall be presented to the Association together with their own.

### ARTICLE VI.

SECTION 1.—*Names of Standing Committees.*—The following Standing Committees of three, shall be appointed at each annual meeting, to prepare, arrange, and expedite business in their several departments for the ensuing year, viz.:

A Committee on Physiology.
" " Dental Pathology and Surgery.
" " Dental Histology and Microscopy.
" " Dental Chemistry.
" " Dental Therapeutics.
" " Operative Dentistry.
" " Mechanical Dentistry.
" " Dental Education.
" " Dental Literature.
" " Prize Essays.

SEC. 2.—*Duties of Members of Committees.*—It shall be the duty of each member of the Standing Committees to make an individual report, so far as such report can be made, and in case of inability to be present at the meeting where that report is due, to forward it to the Recording Secretary, from whom it can be obtained by the chairman of each committee, respectively, at the time of assembling of this Association.

SEC. 3.—*Duties of Committees.*—Each committee shall report, if practicable, the results of original investigations in their several departments, and also such new matter, collected from all the sources at their command, as may be of interest and profit to the Association.

SEC. 4.—*Committees on Operative and Mechanical Dentistry.*—The committees on Operative and Mechanical Dentistry shall thoroughly test, and report upon new modes and materials, and upon their physical properties, stating clearly *why* any particular mode of practice should claim attention, and giving tabulated lists of successes and failures so far as may be obtainable.

SEC. 5.—*Prize Essays.*—Two prizes of medals, not exceeding in value fifty dollars each, may be awarded to the best two communications reported on favorably by the committee on Prize Essays, directed by the Association to be published.

### ARTICLE VII.

*Quorum.*—Fifteen members shall constitute a quorum for the transaction of business in this Association.

### ARTICLE VIII.

*Suspending Rules three-fourths to vote.*—This order of business may be temporarily suspended by a three-fourths vote of all the members present, for the consideration of a specific subject, upon the completion of which the regular order shall be at once resumed.

### ORDER OF BUSINESS.

1st. Meeting of the Executive Committee, filling of vacancies therein, examination of credentials, and payment of dues. 8 A. M.
2d. Organization of the meeting. 10 A. M.
3d. Calling the roll of qualified members.
4th. Reading of the minutes and action thereon.
5th. The reading and consideration of the stated annual reports from the Standing Committees, together with volunteer papers upon the same subjects, in their consecutive order.
6th. Report of Executive Committee.
7th. Balloting for the place of next annual meeting.
8th. Election of Officers, and three members of the Executive Committee.
9th. Appointment of Standing Committees.
10th. Instructions to the Permanent Committees.
11th. Unfinished, new, and Miscellaneous Business.
12th. Adjournment.

### ARTICLE IX.

*Amendments.*—No alteration or amendment shall be made in the foregoing articles, or rules of order, except with the consent of three-fourths of the members present at the subsequent annual meeting to that at which such amendment or alteration may have been proposed in writing.

RULES OF ORDER.

1. On the arrival of the hour of meeting the President shall take the chair, call to order, and announce that the meeting is open for business.

2. No motion or speech shall be in order until the mover or speaker shall have been recognized and assigned the floor by the chair, nor shall a motion be open for debate until seconded and stated by the chair.

3. At the request of any member a motion shall be put in writing.

4. At the request of five members a question shall be divided, or the Yeas and Nays ordered.

5. When a question is under debate no other motion shall be in order, except, 1st, to adjourn; 2d, to lay on the table; 3d, the previous question; 4th, to postpone; 5th, to commit; 6th, to amend; and these motions shall take precedence in the order here stated.

6. The motions to adjourn, to lay on the table and to postpone, shall be decided without debate.

7. A motion to adjourn shall be always in order, but no member can make such a motion while another is speaking, or while a vote or ballot is being taken.

8. A second amendment to the main question shall not be in order until the first is disposed of, nor shall there be an amendment of an amendment to an amendment.

9. After a motion has been seconded and stated by the chair, it shall not be withdrawn without the consent of the Society.

10. No member shall interrupt another while speaking except to call him to order.

11. When called to order a member shall sit down until the point of order is decided by the chair, or in case of appeal by the Society. If the point of order be sustained, the member can proceed in order by the consent of the Society.

12. Every member shall vote upon a question unless excused by the Society.

13. When any motion except to adjourn, has been rejected, it shall not be renewed without unanimous consent.

14. Any member who voted in the majority may move a reconsideration of that question, but if that motion shall be lost, or laid upon the table, it shall not be renewed without unanimous consent.

15. The President may vote with the members upon all questions, but having so voted, shall not give the casting vote in case of a tie.

16. Motions for filling blanks shall be put in the order in which they are moved.

17. On a division, or in voting by Yeas and Nays, any member may change his vote before the result is declared.

18. These rules may be suspended by unanimous consent.

E. A. Bogue,

W. H. Goddard, } *Committee.*

W. H. Morgan,

